# Digital Gaming and Subsequent Health and Well-Being Among Older Adults: Longitudinal Outcome-Wide Analysis

**DOI:** 10.2196/69080

**Published:** 2025-01-27

**Authors:** Atsushi Nakagomi, Kazushige Ide, Katsunori Kondo, Koichiro Shiba

**Affiliations:** 1 Department of Social Preventive Medical Sciences Center for Preventive Medical Sciences Chiba University Chiba Japan; 2 Department of Epidemiology Boston University School of Public Health Boston University Boston, MA United States

**Keywords:** digital gaming, older adults, flourishing, well-being, physical activity, social engagement, mobile phone

## Abstract

**Background:**

Digital gaming has become increasingly popular among older adults, potentially offering cognitive, social, and physical benefits. However, its broader impact on health and well-being, particularly in real-world settings, remains unclear.

**Objective:**

This study aimed to evaluate the multidimensional effects of digital gaming on health and well-being among older adults, using data from the Japan Gerontological Evaluation Study conducted in Matsudo City, Chiba, Japan.

**Methods:**

Data were drawn from 3 survey waves (2020 prebaseline, 2021 baseline, and 2022 follow-up) of the Japan Gerontological Evaluation Study, which targets functionally independent older adults. The exposure variable, digital gaming, was defined as regular video game play and was assessed in 2021. In total, 18 outcomes across 6 domains were evaluated in 2022; domain 1—happiness and life satisfaction, domain 2—physical and mental health, domain 3—meaning and purpose, domain 4—character and virtue, domain 5—close social relationships, and domain 6—health behavior. Furthermore, 10 items from the Human Flourishing Index were included in domains 1-5, with 2 items for each domain. Overall flourishing was defined as the average of the means across these 5 domains. In addition, 7 items related to domains 2, 5, and 6 were assessed. The final sample consisted of 2504 participants aged 65 years or older, with questionnaires containing the Human Flourishing Index randomly distributed to approximately half of the respondents (submodule: n=1243). Consequently, we used 2 datasets for analysis. We applied targeted maximum likelihood estimation to estimate the population average treatment effects, with Bonferroni correction used to adjust for multiple testing.

**Results:**

Digital gaming was not significantly associated with overall flourishing or with any of the 5 domains from the Human Flourishing Index. Although initial analyses indicated associations between digital gaming and participation in hobby groups (mean difference=0.12, *P*=.005) as well as meeting with friends (mean difference=0.076, *P*=.02), these associations did not remain significant after applying the Bonferroni correction for multiple testing. In addition, digital gaming was not associated with increased sedentary behavior or reduced outdoor activities.

**Conclusions:**

This study provides valuable insights into the impact of digital gaming on the health and well-being of older adults in a real-world context. Although digital gaming did not show a significant association with improvements in flourishing or in the individual items across the 5 domains, it was also not associated with increased sedentary behavior or reduced outdoor activities. These findings suggest that digital gaming can be part of a balanced lifestyle for older adults, offering opportunities for social engagement, particularly through hobby groups. Considering the solitary nature of gaming, promoting social gaming opportunities may be a promising approach to enhance the positive effects of digital gaming on well-being.

## Introduction

### Digital Gaming for Health

Digital gaming has become increasingly common among older adults. In 2021, 10.7% of people aged 65 years or older in Japan played digital games for hobbies and entertainment [1]. This trend illustrates the growing appeal of digital gaming among older adults, which holds the potential for promoting both physical and cognitive health [2,3]. A systematic review, for example, has demonstrated that video game–based interventions, such as exergames, can support physical health in older adults by improving balance, mobility, strength, physical fitness, and walking performance [3]. In addition, there is a growing body of evidence that computer games with and without physical components may have positive changes in cognitive function including enhanced executive function, memory, and focused attention [4-6]. Such evidence suggests that digital gaming could serve as a valuable tool for supporting active aging and helping older adults maintain physical and cognitive vitality while providing an enjoyable and engaging activity.

### Limitations of Current Evidence

While existing research has provided valuable insights into the health impacts of digital gaming, several limitations remain. First, although digital games can enhance the lives of older adults in various ways [7], current evidence is largely focused on physical and cognitive health outcomes [3-6,8,9]. The World Health Organization defines health as “a state of complete physical, mental, and social well-being and not merely the absence of disease or infirmity” [10]. This definition highlights the complexity and breadth of health beyond the absence of illness. In recent years, there has been growing recognition of the multidimensional nature of health, leading to increased attention on the concept of human flourishing. Human flourishing refers to “a state in which all aspects of a person’s life are good” and includes five core domains, which are (1) happiness and life satisfaction, (2) physical and mental health, (3) meaning and purpose, (4) character and virtue, and (5) close social relationships [11]. Growing evidence suggests that games can facilitate engaging conversations and serve as a shared activity, helping to promote bridging social capital, foster civic participation, and enhance satisfaction [12,13]. However, the broader social and psychological impacts of digital gaming among older adults remain underexplored [14-16].

Second, much of the existing evidence comes from studies that examine a single or limited number of outcomes, making it difficult to fully understand the range of digital gaming’s effects. The impact of gaming on older adults is inherently multidimensional—potentially beneficial for some outcomes but less so for others. For example, while certain games may encourage physical activity, gaming could also contribute to a more sedentary lifestyle if it predominantly occurs indoors. To gain a holistic understanding of digital gaming’s effects, comprehensive, large-scale studies that assess a wide range of health and well-being outcomes simultaneously are needed.

Third, many previous studies have been conducted in controlled settings, such as short-term exergame interventions lasting 3 months. While these studies provide valuable evidence about the effects of specific gaming interventions, they do not capture the long-term impact of daily gaming activities on health and well-being. To address this gap, studies using well-powered real-world data from community-dwelling older adults are necessary. Such research can offer deeper insights into how regular, everyday digital gaming interacts with the long-term health trajectories of older adults.

### The Aim of This Study

This study aimed to expand the current body of evidence on the multidimensional impact of digital gaming on the human flourishing of older adults. As digital gaming represents a novel and increasingly popular activity among older adults, understanding its associations with holistic health outcomes—including human flourishing—can provide valuable insights into how such activities contribute to their overall well-being and support active aging. We used an outcome-wide analytic approach to simultaneously examine the longitudinal associations between digital gaming and 18 subsequent health and well-being outcomes among Japanese older adults. We examined outcomes across domains of happiness and life satisfaction, physical and mental health, meaning and purpose, character and virtue, social well-being, and health behaviors [17].

## Methods

### Data Sources

**Figure 1:**
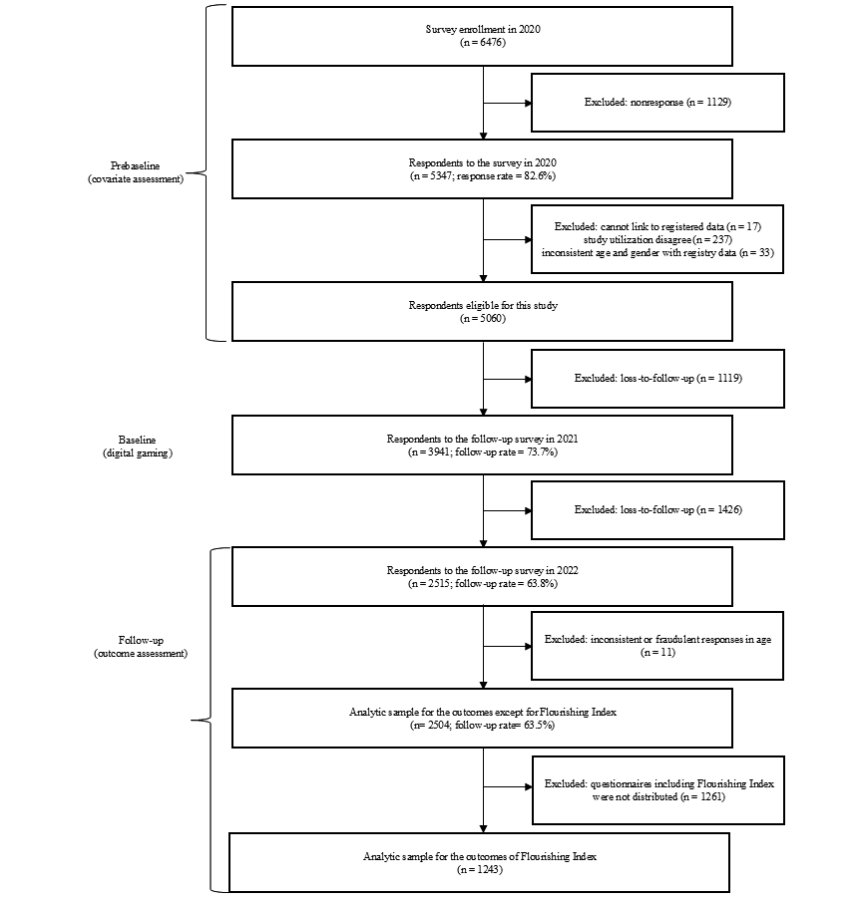
Flowchart of study participants.

We used data from the 2020 (prebaseline), 2021 (baseline), and 2022 (follow-up) waves of the Japan Gerontological Evaluation Study (JAGES), a nationwide survey of adults aged 65 years or older who were not receiving public long-term care insurance benefits in Japan [18]. Our analysis focused on data from participants from one of the JAGES study sites—Matsudo City, as the questionnaire on digital gaming was included only there. Matsudo City is located in Chiba Prefecture, Japan, just northeast of Tokyo. It serves as a suburban area within the Greater Tokyo Area, with a population of around 500,000. Self-administered questionnaires were distributed in 15 districts through random sampling. The study included respondents aged 65 years or older in 2020 who completed the 2020 survey (n=5347; response rate 82.6%). Respondents with inconsistently reported age or gender, as well as those with implausible height (<100 cm or >200 cm) or weight (<30 kg or >100 kg) values, were excluded. Of the 2020 respondents, 73.7% (3941/5060) responded to the 2021 survey. Of the 2021 respondents, 63.8% (2515/3941) responded to the 2022 survey. After excluding 11 participants with inconsistently reported ages, the analytic sample consisted of 2504 participants. For the assessment of human flourishing, the questionnaire version that included the Human Flourishing Index was distributed only to randomly selected half of the respondents (submodule: n=1243). Thus, we used 2 analytic samples—the submodule respondents for flourishing assessments (n=1243) and the full analytic sample for all other outcomes (n=2504). Figure 1 and Figure S1 in Multimedia Appendix 1 provide a detailed flowchart of the data linkage and sample selection process. The participants of the JAGES were informed that their participation was voluntary, and returning the questionnaire implied their consent to participate in the study.

### Measures

We measured the covariates, exposures, and outcomes in a temporal sequence using the 3-wave panel structure of the data (2020, 2021, and 2022). This structure allowed us to account for pre-exposure covariates, including previous values of the outcomes before exposure to digital gaming, which helps mitigate the risks of confounding and reverse causality without the need to adjust for potential mediators [17].

#### Exposure Variable

The exposure variable, digital gaming, was obtained from the 2021 survey. Participants were asked, “Have you played video games with a PC, cell phones, smartphones, tablets, or consoles in the last five years?” The response options included “Playing regularly,” “Have played,” “Know video games, but have not played,” and “Do not know video games.” For analysis, we created a binary variable, where participants who answered “Playing regularly” were coded as 1, indicating regular digital gaming, while all other responses were coded as 0.

#### Outcomes

We examined 18 outcomes in 2022 across 6 domains of health and well-being, following VanderWeele’s human flourishing framework and a previous outcome-wide study using the JAGES data [11,19]. We first assessed human flourishing based on the Human Flourishing Index. Human flourishing refers to “a state in which all aspects of a person’s life are good,” and the Human Flourishing Index is a comprehensive measure designed to capture multidimensional aspects of health and well-being, encompassing several domains [11]. This index consists of 10 self-reported items measuring human well-being outcomes across 5 domains (ie, 2 items per domain). Domain 1 consists of happiness and life satisfaction, domain 2 consists of physical and mental health, domain 3 consists of meaning and purpose, domain 4 consists of character and virtue, and domain 5 consists of close relationships. For each domain, we calculated the mean of the responses to 2 domain-specific items, and overall flourishing was defined as the average of these means across the 5 domains.

In addition to these outcomes, we also examined other outcomes based on items available in the JAGES survey. For domain 2, we assessed instrumental activities of daily living (IADL), forgetfulness, and depressive symptoms using the Geriatric Depression Scale. IADL refers to tasks that enable an individual to live independently within their community, such as managing finances, preparing meals, and handling transportation. For domain 3, we assessed ikigai, broadly defined as “what makes life worth living,” a well-accepted psychological concept in Japanese culture [20]. For domain 5, we assessed participation in hobby groups, participation in sports groups, meeting with friends, and the number of friends. We also examined several health behaviors as outcomes (domain 6: health behavior), including a sedentary lifestyle, smoking, drinking, and frequency of going out. Detailed information on the definitions and measures of these outcomes is shown in Table S1 in Multimedia Appendix 1.

#### Covariates

All covariates were obtained from the 2020 survey, conducted a year before the wave in which the exposure levels were assessed.

As confounders in this study, we included several sociodemographic factors, such as age, gender (men or women), years of education (6-9 years vs 10 or more years), self-rated deprivation, marital status (married or not), living arrangement (living alone or not), and employment status (employed or not).

To minimize the potential for reverse causation, we adjusted for previous values of outcomes wherever available. These included pre-exposure levels of happiness, life satisfaction (as a binary variable), self-rated health, IADL, forgetfulness, Geriatric Depression Scale, participation in hobby groups, participation in sports groups, meeting with friends, number of friends, sedentary behavior, smoking, alcohol consumption, and frequency of going out.

#### Statistical Analysis

This study adopted an outcome-wide analytic approach, which explores the associations between a single exposure and multiple outcomes simultaneously [17,21]. This method allows for a comprehensive assessment of the impact of exposure across a wide range of outcomes. In addition, this approach offers methodological advantages, such as being less susceptible to *P*-hacking and publication bias. It is particularly beneficial for studying the impact of digital gaming, as it captures the multidimensional effects across various domains of health and well-being, providing a holistic understanding of its potential benefits and risks. By examining multiple outcomes within the same framework, this method helps identify patterns and trade-offs that may not be evident when focusing on individual outcomes.

We estimated the population average treatment effects of video gaming on each of the 18 outcomes across the 6 domains, using the doubly robust targeted maximum likelihood estimation. The “doubly robust” method is a statistical approach that combines 2 models—one for the exposure (eg, gaming behavior) and one for the outcome (eg, health or well-being measure). It is termed “doubly robust” because it provides consistent estimates of the average treatment effect as long as at least 1 of these 2 models is correctly specified when the causal identification assumptions are met. Thus, this method is useful as it enhances the robustness of effect estimation by reducing potential biases from model misspecification, compared with methods that rely on a single model (eg, outcome regression and propensity score weighting). To further mitigate the potential bias due to model misspecification, both the exposure and outcome models were fitted data-adaptively using the SuperLearner, an ensemble method that combines weighted estimates from multiple candidate algorithms, including generalized linear models, gradient-boosting machines, and neural networks [22]. The “Super Learner” algorithm is a machine learning technique that data-adaptively selects the optimal weighted combination of estimators from a set of candidate algorithms to improve predictive accuracy. By synthesizing estimates from multiple algorithms, it further reduces the risk of bias due to model misspecification. The targeted maximum likelihood estimation and Super Learning procedures were performed using the ltmle and SuperLearner packages in R (R Foundation for Statistical Computing). For continuous variables, we estimated the difference in the mean levels of the outcomes. All continuous outcomes were standardized (mean 0, SD 1) to ensure that the effect estimates could be interpreted as SD changes in the mean outcomes. For binary outcomes, we estimated risk ratios. To address the issue of multiple testing, we applied the Bonferroni correction that used a highly conservative *P* value threshold for statistical significance of *P*=.00278 (.05/18).

As a sensitivity analysis, we calculated the e-values for each exposure-outcome association to assess the robustness of the estimated associations against unmeasured confounding [23]. E-values quantify the minimum strength of association, on the risk ratio scale, that an unmeasured confounder would need to have with both the exposure and outcome, conditional on the adjusted covariates, for the confounding bias to account for the observed associations.

To address potential bias due to missing data, we used random forest imputation via the R package “missRanger” to impute missing values. All analyses were performed using R (version 4.3.1).

### Ethical Considerations

Ethical approval for the study was obtained from the Ethics Committee at Chiba University (approval M10460). The participants of the JAGES were informed that their participation was voluntary and returning the questionnaire implied their consent to participate in the study.

## Results

Table 1 shows the prebaseline characteristics of the submodule sample by the baseline digital gaming. Among the 1243 respondents, 192 (15.45%) and 380 (15.18%) played digital games, respectively. Individuals who played digital games tended to be younger men, have higher educational attainment, experience less deprivation, be married, live with others, and be employed. These trends were consistent between the submodule sample and the full analytic sample (Table S2 in Multimedia Appendix 1; n=2504).

Table 2 shows the estimated effects of digital gaming on overall flourishing and each of its 5 domains based on the Human Flourishing Index. We found no evidence of the association between digital gaming and these outcomes.

Table 3 shows the estimated effects of digital gaming on other outcomes related to human flourishing and health behaviors. Digital gaming was associated with more frequent participation in hobby groups (mean difference=0.124, *P*=.005) and meeting friends (mean difference=0.076, *P*=.02). However, these associations were above the α=.05 cutoff after applying the Bonferroni correction for multiple testing.

Tables S3 and S4 in Multimedia Appendix 1 present E-values, which indicate the robustness of the observed associations between digital gaming and subsequent health and well-being outcomes against potential unmeasured confounders. For example, in the association between digital gaming and participation in hobby groups, an unmeasured confounder would need to have a risk ratio of at least 1.48 with both the exposure and the outcome, conditional on the measured covariates, to fully account for the observed association. In addition, a risk ratio of 1.22 would be required to shift the CI to include the null value.

**Table 1:** Baseline characteristics of participants who responded to the submodule questionnaires.

Prebaseline characteristics		Digital gaming		
		Total (N=1243)	No (n=1051)	Yes (n=192)
Age (years), mean (SD)		75.7 (5.65)	76.0 (5.68)	74.3 (5.27)
**Gender, n (%)**				
	Men	617 (49.6)	509 (48.4)	108 (56.3)
	Women	626 (50.4)	542 (51.6)	84 (43.8)
**Education, n (%)**				
	High education	1035 (83.3)	865 (82.3)	170 (88.5)
	Low education	208 (16.7)	186 (17.7)	22 (11.5)
**Deprivation, n (%)**				
	No deprivation	1068 (85.9)	892 (84.9)	176 (91.7)
	Deprivation	175 (14.1)	159 (15.1)	16 (8.3)
**Marital status, n (%)**				
	Married	913 (73.5)	754 (71.7)	159 (82.8)
	Not married	330 (26.5)	297 (28.3)	33 (17.2)
**Living alone, n (%)**				
	Living with someone	1026 (82.5)	853 (81.2)	173 (90.1)
	Living alone	217 (17.5)	198 (18.8)	19 (9.9)
**Employment status, n (%)**				
	Employed	309 (24.9)	255 (24.3)	54 (28.1)
	Not employed	934 (75.1)	796 (75.7)	138 (71.9)
Happiness, mean (SD)		7.57 (1.75)	7.56 (1.80)	7.62 (1.49)
**Life satisfaction, n (%)**				
	No	199 (16)	172 (16.4)	27 (14.1)
	Yes	1044 (84)	879 (83.6)	165 (85.9)
**Self-rated health, n (%)**				
	Poor	126 (10.1)	110 (10.5)	16 (8.3)
	Good	1117 (89.9)	941 (89.5)	176 (91.7)
Instrumental activities of daily living, mean (SD)		4.94 (0.355)	4.93 (0.379)	4.97 (0.174)
**Forgetfulness, n (%)**				
	No	1143 (92)	966 (91.9)	177 (92.2)
	Yes	100 (8)	85 (8.1)	15 (7.8)
Depressive symptoms, mean (SD)		2.76 (2.80)	2.82 (2.84)	2.42 (2.55)
Participation in hobby groups, mean (SD)		1.93 (1.39)	1.92 (1.38)	1.98 (1.45)
Participation in sports groups, mean (SD)		2.16 (1.77)	2.11 (1.74)	2.44 (1.91)
Meeting friends, mean (SD)		3.20 (1.65)	3.17 (1.64)	3.37 (1.71)
Number of friends, mean (SD)		2.85 (1.42)	2.81 (1.41)	3.05 (1.45)
**Sedentary lifestyle, n (%)**				
	No	1171 (94.2)	991 (94.3)	180 (93.8)
	Yes	72 (5.8)	60 (5.7)	12 (6.3)
**Smoking, n (%)**				
	No	1143 (92)	972 (92.5)	171 (89.1)
	Yes	100 (8)	79 (7.5)	21 (10.9)
**Alcohol consumption, n (%)**				
	No	672 (54.1)	577 (54.9)	95 (49.5)
	Yes	571 (45.9)	474 (45.1)	97 (50.5)
Going out, mean (SD)		6.12 (1.09)	6.08 (1.11)	6.31 (0.969)

**Table 2:** Digital gaming and subsequent flourishing.

Outcomes	Estimate (95% CI)	*P* value
Flourish (mean across the domains 1-5)	–0.001 (–0.107 to 0.105)	.98
Domain 1 (happiness and life satisfaction)	–0.082 (–0.183 to 0.020)	.12
Domain 2 (mental and physical health)	0.000 (–0.136 to 0.136)	>.99
Domain 3 (meaning and purpose)	–0.001 (–0.119 to 0.117)	.99
Domain 4 (character and virtue)	0.033 (–0.117 to 0.184)	.67
Domain 5 (close social relationship)	0.037 (–0.092 to 0.165)	.58

**Table 3:** Digital gaming and subsequent health and well-being.

Outcomes			Reference (variable type)		Risk ratio or mean difference		
					Estimate (95% CI)		*P* value^a^
**Domain 2 mental and physical health**							
	Instrumental activities of daily living	0.00 (continuous)		0.067 (–0.020 to 0.153)		.13	
	Forgetfulness	1.00 (binary)		1.175 (0.898 to 1.539)		.24	
	Depressive symptoms	0.00 (continuous)		0.027 (–0.039 to 0.093)		.42	
**Domain 3 meaning and purpose**							
	Ikigai	0.00 (continuous)		–0.092 (–0.199 to 0.016)		.09	
**Domain 5 close social relationship**							
	Participation in hobby groups	0.00 (continuous)		0.124 (0.037 to 0.210)		.005^b^	
	Participation in sports groups	0.00 (continuous)		0.011 (–0.053 to 0.075)		.74	
	Meeting with friends	0.00 (continuous)		0.076 (0.010 to 0.142)		.02^c^	
	Number of friends	0.00 (continuous)		0.015 (–0.061 to 0.091)		.70	
**Domain 6 health behavior**							
	Sedentary lifestyle	1.00 (binary)		1.055 (0.788 to 1.411)		.72	
	Smoking	1.00 (binary)		0.991 (0.888 to 1.105)		.87	
	Drinking	1.00 (binary)		1.047 (0.989 to 1.109)		.12	
	Going out	0.00 (continuous)		0.026 (–0.081 to 0.133)		.64	

^a^*P*<.05 after Bonferroni correction (the *P* value cutoff for Bonferroni correction is *P*=.05/18 outcomes [*P*<.00278]).

^b^*P*<.01 before Bonferroni correction.

^c^*P*<.05 before Bonferroni correction.

## Discussion

### Principal Findings

The main findings of this study were threefold. First, video gaming was associated with more frequent participation in hobby groups and meeting with friends, although these results should be interpreted with caution as they may be false positive findings due to multiple testing. Second, video gaming was not associated with lower levels of physical activity, as indicated by measures of sedentary lifestyle and frequency of going out. Third, video gaming did not appear to be associated with the flourishing outcomes across the 5 domains, including psychological well-being.

Our findings highlight the potential of digital gaming to positively impact the social well-being of older adults, contributing to meaningful social interactions and engagement. Previous studies have shown that older adults who play digital games experience social benefits, such as reduced loneliness and improved social functioning [15,24]. However, these studies often relied on smaller samples or qualitative methods, limiting the generalizability of their findings. In contrast, our study, based on a larger sample size, provides more robust evidence to support the social benefits of digital gaming.

Digital gaming may enhance social well-being by fostering participation in hobby groups, where gaming can act as a shared interest and a catalyst for engaging conversations. These interactions create opportunities for older adults to build and strengthen social connections, thereby promoting a sense of belonging and community. This underscores the role of digital gaming as not only a recreational activity but also a potential tool for enhancing social engagement and overall well-being among older adults.

Digital gaming, particularly nonphysical types, may reduce physical activity levels among older adults by promoting sedentary behavior, as players often remain seated for extended periods. This sedentary time could potentially replace activities like walking or outdoor socializing. In contrast, exergames—digital games that require physical movement—have been shown to promote physical activity by improving mobility, balance, and fitness levels [2]. Despite concerns about sedentary behavior, our study found no evidence in a real-world context that digital gaming was associated with increased sedentary behavior or decreased outdoor activities among older adults in Japan. This finding suggests that, for many older adults, digital gaming can integrate into a balanced lifestyle without negatively affecting physical activity levels.

Digital gaming may also enhance psychological well-being among older adults by providing relaxation and social interaction [24,25], although we did not find evidence of such improvements in our study. Research indicates that the social aspects of gaming, such as playing together or discussing games with family and friends, are crucial for translating digital gaming experiences into positive well-being outcomes [15,26]. However, many older adults tend to play digital games alone, which can limit the potential social benefits that gaming might otherwise offer. This highlights the need for targeted initiatives to promote and encourage more social forms of digital gaming among older. Facilitating environments where digital games are played together, such as community centers or online platforms designed for older users, could help harness the social benefits of gaming and, in turn, improve their overall well-being.

This study has several limitations. First, the broad definition of gaming behavior used in this study may not adequately reflect the specific characteristics and impacts of individual games. The diversity of digital games, including their types, purposes, and contexts, means that our analysis may have attenuated potential associations. In addition, whether participants played alone or with others could significantly alter the associations. Future research should adopt a more nuanced approach by categorizing gaming behavior to better capture the specific effects of different game types.

Second, the generalizability of our findings is limited, as our data were derived from a single city in Japan. Results may differ in other regions or countries with different cultural contexts and gaming habits.

Third, due to the observational nature of this study, the possibility of unmeasured confounding and reverse causation cannot be completely ruled out. However, to mitigate this issue, we adjusted for numerous covariates, including confounders and prebaseline outcomes, as recommended in the outcome-wide approach [17,21]. Fourth, while our study is larger than previous studies, it is still likely underpowered (eg, n=1243 with an exposure prevalence of approximately 15%). Future studies with larger sample sizes are warranted to provide more robust conclusions.

Fifth, the outcome-wide analytic approach used in this study was designed to provide a comprehensive overview of associations across multiple outcomes; however, it does indeed sacrifice depth for breadth due to the word count limit. The traditional design focusing on a single or a narrow set of outcomes has other limitations (eg, not providing holistic evidence on gaming-health associations, *P*-hacking, and publication bias), which the outcome-wide design seeks to address, at least to some extent. We think this is a trade-off, and the outcome-wide design (which contains more breadth) and traditional study design (which contains more in-depth discussion) are both complementary and play different roles in the advancement of science. Future research on specific outcomes with more detailed analysis (eg, effect heterogeneity across subgroups) is needed.

Finally, our study did not account for the potential overlap between digital gaming and social media usage. Gamers are more likely to use social media, and the impacts of gaming may overlap with those of social media usage. This overlap is particularly relevant as many digital games include social interaction features that resemble social media functions, such as in-game chats or multiplayer platforms. Consequently, some observed associations may reflect the combined effects of gaming and social media rather than gaming alone. Future studies should measure and differentiate these activities to clarify their unique and overlapping impacts on health and well-being.

### Conclusion

While digital gaming did not show a significant association with improvements in flourishing or its subdomains, our results indicate that digital gaming could lead to greater social engagement without increasing sedentary behavior and reducing outdoor activities. These results suggest that digital gaming can be integrated into a balanced lifestyle, offering a potential tool for enhancing social well-being in older adults. Furthermore, integrating digital gaming into community and health care settings presents novel opportunities for fostering social connections and supporting healthy aging in this population.

## Appendix

Multimedia Appendix 1 Supplementary data.

## Abbreviations

JAGES: Japan Gerontological Evaluation Study
IADL: instrumental activities of daily living

## Data Availability

The datasets analyzed during this study are available from the corresponding author on reasonable request.
