# Supplementary material for: Digital Gaming and Subsequent Health and Well-Being Among Older Adults: Longitudinal Outcome-Wide Analysis
Source: J Med Internet Res. 2025 Jan 27;27:e69080. doi: 10.2196/69080 (PMC11811670; doi:10.2196/69080)
Supplement: Multimedia Appendix 1 [file jmir_v27i1e69080_app1.docx]

**Figure S1.** Diagram illustrating the temporal order of data linkage and variables included in the analysis


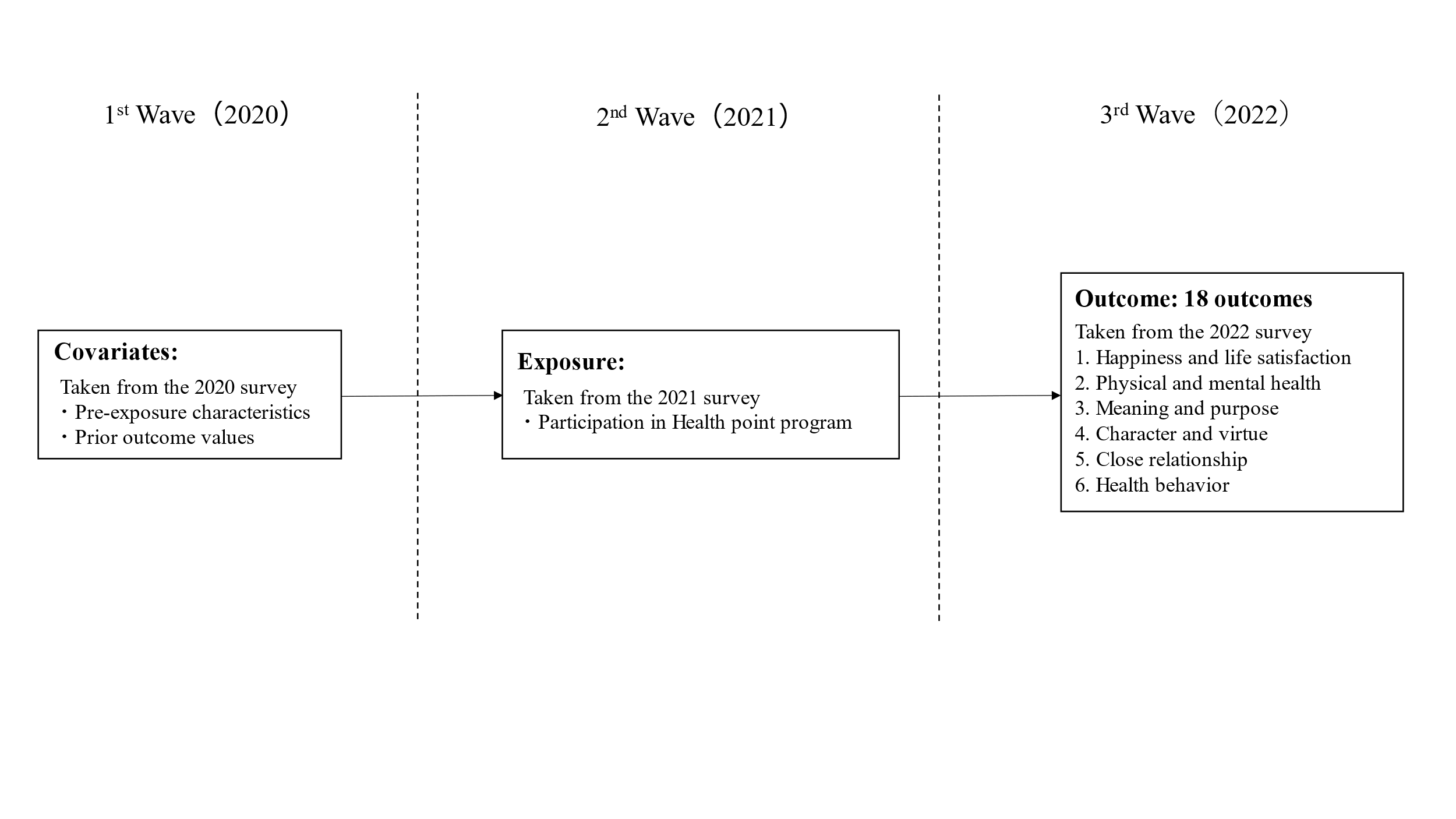


**Table S1.** Definition of outcome variables.

| Domain | Outcomes | Outcome definition and questions |
| --- | --- | --- |
|  | Flourishing Index | Continuous (standardized): Means of Happiness and life satisfaction, Mental and Physical health, Meaning and Purpose, Character and Virtue, and Close Social Relationship (0-10). |
| 1. Happiness and life satisfaction | | |
|  | Happiness and life satisfaction | Continuous (standardized): Means of happiness (0-10) and life satisfaction (0-10).  Questions and options: In general, how happy or unhappy do you usually feel? 0 = Extremely Unhappy, 10 = Extremely Happy Overall, how satisfied are you with life as a whole these days?  0 = Not Satisfied at All, 10 = Completely Satisfied |
| 2. Mental and Physical health | | |
|  | Mental and Physical health | Continuous (standardized): Means of self-rated mental health (0-10) and physical health (0-10).  Questions and options: In general, how would you rate your physical health? 0 = Poor, 10 = Excellent How would you rate your overall mental health? 0 = Poor, 10 = Excellent |
|  | Instrumental activities of daily living | Continuous (standardized): Sum of the 5 items.  Questions: 1. Can you go out alone by train or bus? 2. Can you go shopping for daily necessities? 3. Can you cook for yourself? 4. Can you pay your bills by yourself? 5. Can you deposit or withdraw money from your bank/postal savings account(s) by yourself?  Options: 1. Yes (or Yes but I usually don’t) 0. No |
|  | Forgetfulness | Binary (1=forgetfullness vs 0=non-forgetfullness)  Question: Do people around you say that you often forget things, such as asking the same question repeatedly?  Options: 1. Yes 0. No |
|  | Depressive symptoms | Continuous (standardized):  The Japanese version of Geriatric Depression Scale (15 items), ranges 0-15, greater values indicate more depressive symptoms |
| 3. Meaning and Purpose | |  |
|  | Meaning and Purpose | Continuous (standardized): Means of meaning of life (0-10) and purpose in life (0-10).  Questions and options: Overall, to what extent do you feel the things you do in your life are worthwhile? 0 = Not at All Worthwhile, 10 = Completely Worthwhile I understand my purpose in life. 0 = Strongly Disagree, 10 = Strongly Agree |
|  | Ikigai | Continous (standardized): Ikigai (0-10)  Questions and options: Do you have ikigai? 0 = Strongly Disagree, 10 = Strongly Agree |
| 4. Character and Virtue | |  |
|  | Character and Virtue | Continuous (standardized): Means of integrity (0-10) and delayed gratification (0-10).  Questions and options: I always act to promote good in all circumstances, even in difficult and challenging situations. 0 = Not True of Me, 10 = Completely True of Me I am always able to give up some happiness now for greater happiness later. 0 = Not True of Me, 10 = Completely True of Me |
| 5. Close Social Relationship | |  |
|  | Close Social Relationships | Continuous (standardized): Means of Content with relationship (0-10) and satisfaction of relationship (0-10).  Questions and options: I am content with my friendships and relationships. 0 = Strongly Disagree, 10 = Strongly Agree My relationships are as satisfying as I would want them to be. 0 = Strongly Disagree, 10 = Strongly Agree |
|  | Participation in hobby groups | Continuous (standardized)  Question: How often do you attend activities for the following groups – Hobby groups  Options: 1. 4 or more times a week 2. 2 -3 times a week 3. Once a week 4. 1-3 times a month 5. A few times a year 6. Never |
|  | Participation in sports groups | Continuous standardized)  Question: How often do you attend activities for the following groups – Sports groups  Options: 1. 4 or more times a week 2. 2 -3 times a week 3. Once a week 4. 1-3 times a month 5. A few times a year 6. Never |
|  | Frequency of meeting friends | Continuous (standardized)  Question: How often do you see your friends?  Options: 1. 4 or more times a week 2. 2 -3 times a week 3. Once a week 4. 1-3 times a month 5. A few times a year 6. Hardly/None |
|  | Number of friends seen within a month | Continuous (standardized)  Question: How many friends/acquaintances have you seen over the past month? Count the same person as one, no matter how many times you have seen him/her.  Options: 1. None 2. 1-2 3. 3-5 4. 6-9 5. 10 or more |
| 6. Health behavior | |  |
|  | Sedentary lifestyle | Binary (1 = sedentary lifestyle, 0 = non-sedentary lifestyle): Sedentary lifestyle was defined as sitting for eight hours a day or longer AND engaging in vigorous physical activities < 1hour/day AND walking or sitting < 3hour/day  Question:  How long do you usually spend time during a day doing the following activities?  Options: • Works and sports involving vigorous physical activities (1 = none, 2 = less than one hour, 3 = 1 hour or longer) • Sitting (1 = less than three hours, 2= three hours to less than eight hours, 3 = eight hours or longer) • Walking or standing (1 = less than one hour, 2 = one hour to less than three hours, 3 = three hours or longer) |
|  | Current smoking status | Binary (1=current smoker vs. 0=non-smoker)  Question:  Do you smoke cigarettes?  Options 1. I smoke almost every day. 2. I sometimes smoke. 3. I quit smoking within 5 years and do not smoke now. 4. I quit smoking more than 5 years ago and do not smoke now. 5. I never smoke. |
|  | Drinking | Binary (1=current drinking vs. 0=non-drinking)  Question:  Do you drink alcohol?   Options: 1. I drink almost every day. 2. I sometimes drink. 3. I quit drinking within 5 years and do not drink now. 4. I quit drinking more than 5 years ago and do not drink now. 5. I never drink. |
|  | Frequency of going out | Continuous (standardized)  Question:  How often do you go out?  Options: 1. 4 or more a week 2. Two or three times a week 3. Once a week 4. One to three times a month 5. Several times a year 6. Rarely |

**Table S2.** Baseline characteristics of participants who responded to the entire questionnaires.

|  |  | Digital gaming | |
| --- | --- | --- | --- |
| **Pre-baseline characteristics** | **Total** | **No** | **Yes** |
|  | **(N=2504)** | **(N=2124)** | **(N=380)** |
| **Age (years)** |  |  |  |
| Mean (standard deviation) | 75.7 (5.59) | 76.0 (5.60) | 74.1 (5.22) |
| **Gender** |  |  |  |
| Men | 1212 (48.4%) | 1012 (47.6%) | 200 (52.6%) |
| Women | 1292 (51.6%) | 1112 (52.4%) | 180 (47.4%) |
| **Education** |  |  |  |
| High education | 2112 (84.3%) | 1773 (83.5%) | 339 (89.2%) |
| Low education | 392 (15.7%) | 351 (16.5%) | 41 (10.8%) |
| **Deprivation** |  |  |  |
| No deprivation | 2132 (85.1%) | 1792 (84.4%) | 340 (89.5%) |
| Deprivation | 372 (14.9%) | 332 (15.6%) | 40 (10.5%) |
| **Marital status** | |  |  |
| Married | 1836 (73.3%) | 1533 (72.2%) | 303 (79.7%) |
| Not married | 668 (26.7%) | 591 (27.8%) | 77 (20.3%) |
| **Living alone** |  |  |  |
| Living with someone | 2086 (83.3%) | 1748 (82.3%) | 338 (88.9%) |
| Living alone | 418 (16.7%) | 376 (17.7%) | 42 (11.1%) |
| **Employment status** | |  |  |
| Employed | 631 (25.2%) | 518 (24.4%) | 113 (29.7%) |
| Not employed | 1873 (74.8%) | 1606 (75.6%) | 267 (70.3%) |
| **Happiness** |  |  |  |
| Mean (standard deviation) | 7.54 (1.76) | 7.52 (1.79) | 7.66 (1.56) |
| **Life satisfaction** | |  |  |
| No | 436 (17.4%) | 380 (17.9%) | 56 (14.7%) |
| Yes | 2068 (82.6%) | 1744 (82.1%) | 324 (85.3%) |
| **Self-rated health** | |  |  |
| Poor | 236 (9.4%) | 209 (9.8%) | 27 (7.1%) |
| Good | 2268 (90.6%) | 1915 (90.2%) | 353 (92.9%) |
| **Instrumental activities of daily living** | | |  |
| Mean (standard deviation) | 4.93 (0.366) | 4.93 (0.382) | 4.96 (0.264) |
| **Forgetfulness** | |  |  |
| No | 2301 (91.9%) | 1949 (91.8%) | 352 (92.6%) |
| Yes | 203 (8.1%) | 175 (8.2%) | 28 (7.4%) |
| **Depressive symptoms** | |  |  |
| Mean (standard deviation) | 2.83 (2.90) | 2.88 (2.92) | 2.60 (2.74) |
| **Participation in hobby groups** | | |  |
| Mean (standard deviation) | 1.90 (1.37) | 1.89 (1.37) | 1.96 (1.40) |
| **Participation in sports groups** | | |  |
| Mean (standard deviation) | 2.16 (1.76) | 2.11 (1.73) | 2.45 (1.90) |
| **Meeting friends** | |  |  |
| Mean (standard deviation) | 3.16 (1.65) | 3.16 (1.64) | 3.20 (1.68) |
| **Number of friends** | |  |  |
| Mean (standard deviation) | 2.80 (1.42) | 2.78 (1.42) | 2.89 (1.46) |
| **Sedentary lifestyle** | |  |  |
| No | 2387 (95.3%) | 2031 (95.6%) | 356 (93.7%) |
| Yes | 117 (4.7%) | 93 (4.4%) | 24 (6.3%) |
| **Smoking** |  |  |  |
| No | 2315 (92.5%) | 1976 (93.0%) | 339 (89.2%) |
| Yes | 189 (7.5%) | 148 (7.0%) | 41 (10.8%) |
| **Alcohol consumption** | |  |  |
| No | 1361 (54.4%) | 1179 (55.5%) | 182 (47.9%) |
| Yes | 1143 (45.6%) | 945 (44.5%) | 198 (52.1%) |
| **Going out** |  |  |  |
| Mean (standard deviation) | 6.12 (1.09) | 6.09 (1.11) | 6.27 (0.981) |

**Table S3.** Robustness to unmeasured confounding (E-Values) of associations between digital gaming and flourish.

| Outcomes |  | Estimate | CI limit |
| --- | --- | --- | --- |
| Flourish (Mean across the domains 1 - 5) | | 1.033 | 1.000 |
| Domain 1 Happiness and Life satisfaction | | 1.365 | 1.000 |
| Domain 2 Mental and Physical health | | 1.019 | 1.000 |
| Domain 3 Meaning and Purpose | | 1.032 | 1.000 |
| Domain 4 Character and Virtue | | 1.208 | 1.000 |
| Domain 5 Close social relationship | | 1.221 | 1.000 |
| CI: confidence interval | |  |  |

**Table S4.** Robustness to unmeasured confounding (E-Values) of associations between digital gaming and health and well-being.

| Outcomes |  | Estimate | Lower CI |
| --- | --- | --- | --- |
| Domain 2 Mental and Physical health | |  |  |
|  | Instrumental activities of daily living | 1.321 | 1.000 |
|  | Forgetfulness | 1.629 | 1.000 |
|  | Depressive symptoms | 1.185 | 1.000 |
| Domain 3 Meaning and Purpose | |  |  |
|  | Ikigai | 1.395 | 1.000 |
| Domain 5 Close social relationship | |  |  |
|  | Participation in hobby groups | 1.484 | 1.223 |
|  | Participation in sports groups | 1.109 | 1.000 |
|  | Meeting with friends | 1.349 | 1.105 |
|  | Number of friends | 1.133 | 1.000 |
| Domain 6 Health behavior | |  |  |
|  | Sedentary lifestyle | 1.295 | 1.000 |
|  | Smoking | 1.108 | 1.000 |
|  | Drinking | 1.270 | 1.000 |
|  | Going out | 1.181 | 1.000 |
| CI: confidence interval | |  |  |
